# Determinants of breastfeeding practice in Pujehun district, southern Sierra Leone: a mixed-method study

**DOI:** 10.1186/s13006-021-00390-4

**Published:** 2021-05-26

**Authors:** Dorothee van Breevoort, Francesca Tognon, Arne Beguin, Amara S. Ngegbai, Giovanni Putoto, Ankie van den Broek

**Affiliations:** 1 Doctors with Africa CUAMM, Pujehun-Freetown, Sierra Leone; 2 Doctors with Africa CUAMM, Via San Francesco, 126, 35121 Padua, Italy; 3 Department of Women’s and Children’s Health, University of Padua, Padua, Italy; 4 MoHS Sierra Leone, Pujehun District, Sierra Leone; 5 Department of Global Health, Royal Tropical Institute (KIT), Amsterdam, The Netherlands

**Keywords:** Breastfeeding, Determinants, Sierra Leone, Child health, Mix-method

## Abstract

**Background:**

It is well established that exclusive breastfeeding can play a critical role in reducing child morbidity and mortality. Limited research has been done thus far on the practice and perceptions of breastfeeding in Sierra Leone, where more than 10 % of children die before the age of five. This study aimed to gain understanding into and explore both matters in order to develop recommendations for effective strategies to promote breastfeeding practice in Pujehun District, Southern Sierra Leone.

**Methods:**

This exploratory mixed-method study included a cross-sectional survey of 194 mothers, semi-structured interviews and focus group discussions. Logistic regression analysis was used calculated odds ratios of factors associated with primarily breastfeeding practice, defined as ‘Children under six months of age who are fed with breast milk only and children older than six months of age that were exclusively breastfed up to six months’, based on recall from birth. Exclusive breastfeeding rate was based on breastfeeding practice 24 h prior to the survey. Qualitative data was analysed through a deductive approach, using a pre-determined framework on determinants of breastfeeding.

**Results:**

This study revealed an exclusive breastfeeding rate of 62.8% (95% CI 53.9, 71.7); dropping from 74% in the 0–1-month age group to 33% in the 4–5 months group. Triangulation of qualitative and quantitative data revealed enabling factors for primarily breastfeeding practice included mothers receiving support during their first breastfeed, pregnant women being provided with information on the benefits of the practice, counselling by nurses, support from husbands, and women’s awareness of how their friends and family members fed their own babies. The main barriers were a lack of encouragement by husbands, women’s perception that their infants’ stools were abnormal or that they were not producing enough breast milk.

**Conclusions:**

Although the exclusive breastfeeding may have risen over recent years, a gap remains compared to World Health Organization recommendations. According to the breastfeeding determinants identified in this study, promotion of counselling by a nurse, encouragement of husbands’ support, and improve knowledge of mothers on breastfeeding are recommended to be incorporated in the design of future health programs.

## Background

Breastfeeding has major benefits for infants’ and young children’s health and survival [1]. It has been shown that exclusive breastfeeding (EBF) reduces morbidity and mortality due to infectious diseases, with a particular protective effect against diarrhea, respiratory infections and otitis media; moreover, breastfed infants have a lower risk of hospital admission [1, 2]. Exclusive breastfeeding is defined as “the infant receives breastmilk and allows the infant to receive ORS, drops, syrups but nothing else” [3]. The EBF rate is based on feeding practice 24 h prior the assessment [4]. It has been established that EBF for the first 6 months of life and continued breastfeeding with adequate complementary foods up to the age of 2 years or beyond, can play a critical role in reducing child mortality, saving the lives of over 820,000 children below the age of 5 years worldwide, every year [2, 5].

Despite the recommendations of the World Health Organization (WHO) that infants be exclusively breastfed for the first 6 months of life [6], it is estimated that only 36% of infants under 6 months old were exclusively breastfed worldwide in the 2007–14 period [5]. In 2017, the number rose only slightly, to 40% [7].

Sierra Leone has one of Africa’s highest rates of child mortality: in 2013, the country’s infant mortality rate was 92 deaths per 1000 live births and its under-five mortality rate was 156 deaths per 1000 live births [8]. Exclusive breastfeeding rates have risen in the country in recent years: SLDHS 2019 showed an increase from 32% in 2013 to 54% in 2019 [8, 9], and the 2017 nutrition survey showed an EBF rate of 61.6% for Pujehun District [10]. However, despite the expansion of EBF in recent years and improvement in under-five mortality, which fell to 94 per 1000 live births in 2017 [11], there are still opportunities to improve breastfeeding practice. It is essential that policymakers understand the determinants underlying the practice in order to design appropriate and effective interventions aimed at improving child health.

Studies have been conducted worldwide, including in several low- and middle-income countries, to gain understanding into the determinants that contribute to breastfeeding practices. Such factors include women’s attending antenatal care and postnatal care services [12], caregivers’ knowledge about breastfeeding [13], the influence of family and community members [13, 14], and the burden of other responsibilities [13]*.* Thus, the range of determinants contributing to breastfeeding practices in low-resource settings is wide. However, only limited research has been conducted thus far to investigate the social determinants of breastfeeding in Sierra Leone. Sharkey et al. found that some mothers believe their infants’ frequent stools are an indication that breast milk is harmful to them [15]. Some are convinced that having sex while breastfeeding will contaminate their breast milk [15]. Further research needs to be done to gain in-depth knowledge of the determinants of EBF in Sierra Leone, so as to be able to devise appropriate strategies to improve the practice. The aim of this study was to understand current breastfeeding practice and perceptions in Sierra Leone, and more specifically in Pujehun District.

## Methods

### Study setting

Pujehun is a rural district in the Southern Province of Sierra Leone. It has 346,461 inhabitants, including an estimated under-five population of 56,990 and an estimated 15,244 pregnant women every year, according to the most recent population report (2015 [16];). The district is divided into 12 administrative subdivisions known as chiefdoms. Data from 2017 showed that 98.8% of the district’s women had attended at least one antenatal care visit, and 90.9% had delivered their babies in a health facility. Immunization coverage of pentavalent 3 and Oral Polio Vaccination (OPV) 3 was 99.2 and 96.5%, respectively [11].

### Study design

We conducted an exploratory mixed-methods study. A cross-sectional survey, semi-structured interviews (SSIs) and Focus Groups Discussions (FGDs) were carried out from May to July 2018 in Pujehun District, Sierra Leone.

### Sampling methods and data collection

The survey involved 194 mothers of children younger than 24 months of age. The sample size was calculated based on a confidence level of 95% and a margin of error of 10%, an expected prevalence of 0.5 (for social determinants, since there is no standard prevalence for social determinants*)* and a design effect of two. The respondents were selected during their routine visits to health posts in five purposely selected chiefdoms, the latter having been selected based on their geographical variation (physical accessibility) and variation in users’ health-seeking behavior (percentage of institutional deliveries).

Our survey was conducted at the facility level during routine under-five clinic days for the vaccination and growth-monitoring of children. Mothers of children under 2 years of age were randomly selected and asked to participate in the study. Inclusion criteria for the mother-child pair was that woman is the biological mother from a child and the mother is 16 years or above. One health center was purposely selected in each of the five chiefdoms based on the size of the target population*.* We interviewed 113 mothers of children under 6 months of age and 81 mothers of children aged from six to 23 months. The questionnaire was adapted from a validated questionnaire to assess breastfeeding intentions and practice in Nigeria [17]. The interviewer administered pretested questionnaire requesting information on social demographics, obstetric factors, breastfeeding practice and breastfeeding-related factors. The survey was administered in the respondents’ local language (Mende or Krio).

In a convergent parallel approach, SSI were conducted with mothers of children under 2 years and with healthcare workers (HCW). Twenty mothers were purposely selected based on their breastfeeding practices and asked to participate in an SSI after completing the questionnaire in the five health centers. The SSI was used to collect more in-depth information on breastfeeding-related factors e.g., reasons behind the decision to continue or stop EBF and factors influencing the mothers in decision making regarding breastfeeding practice. Participants were selected based on their practice of EBF for children under the age of 6 months (five mothers), non-EBF of children under the age of 6 months (10 mothers), and mothers of children aged six to 23 months (five mothers) In addition, six HCW were purposely chosen from each of the selected health facilities for an SSI in order to collect data regarding breastfeeding at the health-system level. Questions were related to the role of the HCW regarding EBF, knowledge and awareness of tools and guidelines for HCW and the reason of women to stop or continue EBF according to the Health Care Worker. The HCW interviews were conducted in English and audio-recorded, with notes being taken contemporaneously*.*

In July 2018, a sequential mixed-method approach was introduced with the use of FGDs to delve deeper into the findings of the questionnaires and the SSIs. Seven FGDs were conducted with mothers of children under the age of 2 years (two groups of 7 to 8 women), fathers (two groups of 9 men) and community members (two groups of 9 to 11 people). Participants were purposely selected based on the criteria of the groups (mothers, fathers, community members), and asked by the research assistant to attend the Focus Groups Discussions. Focus Groups Discussions were conducted at two communities where questionnaires had previously been administered. In addition, one FGD was conducted with six Health Care Workers. All of the FGDs were conducted in the Mende language except for the HCW FDG, which was conducted in English. Each interview and FGD was audio-recorded, and notes were taken during the Focus Group Discussions.

### Data analysis

The questionnaire data was entered into a Kobo toolbox database and systematically analyzed using Epi-Info 7, MS Excel, and SPSS statistics 25. As part of the data-quality assurance procedure, the main investigator double-checked all of the data entry and a second member of the research team cross-checked a sample of the data. The statistical analyses of the EBF rate were based on a 24-h recall including the 113 participants (mothers of children under 6 months of age). Potential determinants revealed by the survey were investigated for correlation with breastfeeding practice. The statistical analysis of the determinants was based on ‘recall from birth’. These analyses included all 194 participants. Simple logistic regression analysis was used to calculate crude odds ratio (COR) and 95% confidence interval (CI). All covariates associated with outcome variable were included in a multiple logistic regression analysis to assess the association of independent variable with the outcome variable. Adjusted odds ratio (AOR) and 95% CI was used to measure the strength of the independent association. A *P*-value less than 0.05 was considered as significant. The audio recordings of the SSIs and FGDs were transcribed and translated into English by a multilingual (Krio Mende and English) speaker. Analysis of the transcript was based on the deductive content analysis approach. The data was coded and systematically analyzed after being entered into an Excel spreadsheet.

### Definitions

#### Exclusive breastfeeding

Infants under 6 months of age who are fed exclusively with breast milk and allows the infant to receive ORS, drops, syrups but nothing else, based on 24-h recall.

#### Early initiation

Children born in the last 24 months who were put to the breast within 1 hour of birth based on recall from birth.

#### Primarily breastfeeding

Children under 6 months of age who are fed with breast milk only and children older than 6 months of age that were exclusively breastfed up to 6 months, based on recall from birth.

## Results

### Overview of participants

A total of 194 mothers of children under the age of 2 years responded to the interviewer-administered questionnaire, including 113 mothers of infants under 6 months of age and 81 mothers of children age six to 23 months. The mean age (± SD) of the participants was 24.5 (6.6) years, with a range of 16 to 48 years. Of the mothers interviewed 43% reported not to have received education, while 35% attended secondary education. The majority of the mothers were self-employed (56%) (e.g., farmer or trader), while 8% were student (secondary school). The vast majority of the mothers were married or had a partner (94%). Fifty-three of the women (27.3%) who brought a child they came with to clinic was their first child (primipara). The range of multipara was 2–11 births. The mean age (± SD) of the infants was 6 months (5.4) with a range of 2 days to 23.9 months. These and other social demographic characteristics are shown in Table 1.

**Table 1:** Social demographic characteristics of mothers with children under the age of 2 in Pujehun District

		Mothers of child < 6 months	Mothers of Child 6–23 months	Total
Variable		n (%)	n (%)	n (%)
No. of participants		113 (58.2)	81 (41.8)	**194 (100)**
Age of mother	16–19	30 (28.3)	18 (23.7)	**48 (26.4)**
	20–25	44 (41.5)	27 (35.5)	**71 (39.1)**
	26–30	17 (16.0)	20 (26.0)	**37 (20.3)**
	31–35	5 (4.7)	5 (6.5)	**10 (5.5)**
	> 35	10 (9.4)	6 (7.8)	**16 (8.8)**
Age of mother (mean, DS)		24.2 (7.0)	24.9 (5.9)	**24.5 (6.6)**
Mother’s educational level	No education	48 (42.5)	35 (43.2)	**83 (42.7)**
	Primary education	24 (21.2)	16 (19.8)	**40 (20.6)**
	Secondary education	39 (34.5)	30 (37.0)	**69 (35.16)**
	Tertiary education	2 (1.8)	0	**2 (1.0)**
Mother’s occupation	Employed	3 (2.7)	0	**3 (1.6)**
	Housewife	42 (37.2)	18 (22.2)	**60 (30.9)**
	Self-employed	57 (50.44)	52 (64.2)	**109 (56.2)**
	Student	7 (6.19)	9 (11.1)	**16 (8.3)**
	Other	4 (3.5)	2 (2.5)	**6 (3.1)**
Marital status	Married/partner	105 (92.9)	78 (96.3)	**183 (94.3)**
	Single	8 (7.1)	3 (3.7)	**11 (5.7)**
Parity	Primipara	30 (26.5)	23 (28.4)	**53 (27.3)**
	Multipara (2–4)	64 (56.6)	39 (48.1)	**103 (53.1)**
	Grand multipara (> 4–11)	19 (16.8)	19 (23.4)	**38 (19.6)**
Number of children delivered (mean, SD)	3.0 (2.1)	2.9 (2.1)	3.1 (1.0)	**3.0 (2.1)**
Children alive (mean, SD)	2.4 (1.5)	2.4 (1.5)	2.5 (1.5)	**2.4 (1.5)**
Sex of child	Male	60 (53.1)	41 (50.6)	**101 (52.1)**
	Female	53 (46.9)	40 (49.4)	**93 (47.9)**
Age of child in months (mean, SD)		2.4 (1.6)	11.7 (4.7)	**6.0 (5.4)**

Table 2 shows the main maternal health related characteristics. The vast majority of the mothers received antenatal care at least once (193; 99.5%) and 66.4% (129) had received postnatal care. Most of the mothers (182; 94%) had received information about the feeding of children prior to giving birth, although not all of those who received such information reported having also received information on the benefits of breastfeeding (177; 91%). All but two of the women reported having had a facility-based delivery (either hospital or health center). Of those who reported problems with breastfeeding (22), 18 (9% of all of the mothers) said they experienced nipple pain.

**Table 2:** Maternal health-related characteristics of mothers with children under the age of 2 in Pujehun District

Variable		Number	%
ANC^a^ services attended	Yes	**193**	99.5
	No	**1**	0.5
PNC^b^ services attended	Yes	**129**	66.5
	No	**65**	33.5
Receiving information during pregnancy on feeding	Yes	**182**	93.8
	No	**12**	6.2
Information on health benefits of breastfeeding received during pregnancy	Yes	**177**	91.2
	No	**17**	8.8
Place of delivery	Health center	**138**	71.1
	Hospital	**54**	27.8
	Home	**2**	1.0
Mode of delivery	Vaginal	**175**	90.2
	Caesarean section	**19**	9.8
Initiation of breastfeeding (from time of delivery)	Within one hour	**139**	71.6
	From 1 to 24 h	**47**	24.2
	More than 24 h	**8**	4.1
Assisted by HCW during first breastfeeding	Yes	**147**	75.8
	No	**47**	24.2
Difficulties with breastfeeding	No	**172**	88.7
	Mastitis	**4**	2.1
	Nipple pain	**18**	9.3

^a^ Antenatal Care

^b^ Postnatal Care

### Breastfeeding practices among respondents

WHO has developed several indicators to assess infant and young child feeding practice, among other, early initiation of breastfeeding, EBF under 6 months, and children ever breastfed [3]. Of all the mothers included in the study, 71.6% (95% CI 65.2, 78.1) reported having initiated breastfeeding within the first hour after birth.

Of the 113 mothers of infants under 6 months of age, 71 reported having fed breastmilk alone in the previous 24 h, given an EBF rate of 62.8% (95% CI 53.9, 71.7) based on a 24-h recall. Disaggregating the data into smaller age groups revealed that of the infants within the 0–1-month group, 74% were exclusively breastfed. EBF decreased in the 2–3 months (66%) and 4–5 months (33%) age groups. There was no significant difference in EBF rates among the five health centers.

All of the mothers surveyed (194, 100%) reported having breastfed their child at least once.

### Factors associated with breastfeeding practices

#### Age and parity of the mother

The age of mothers is significantly associated with primarily breastfeeding. While 73% (106/146) of the mothers 20 years or older practiced primarily breastfeeding, only 42% (20/48) of teenage mothers (16–19 years of age) did so (COR 0.3; 95% CI 0.1, 0.5, *p* < 0.001, Table 3). Focus Group Discussion participants mentioned several possible reasons as to why some teenage mothers do not practice EBF, including their need to attend school, their belief that if they do so “the breast will slack”, and time constraints due to competing activities e.g., social networking with other adolescence.

**Table 3:** Determinants associated with primarily breastfeeding based on logistic regression

Variable (*n* = 194)	Total (*n*)	Primarily BF *n* (%)	Non-primarily BF (%)	COR (95% CI)	AOR (95% CI)
Age					
< 20 years old (teenager)	48	20 *(42)*	28 *(58)*	0.3 (0.1,0.5)***	0.5 (0.2,1.3)
≥ 20 years	146	106 (*73*)	40 (*27*)	1	1
Parity					
(Grand) multipara	141	102 (*72*)	39 (*28*)	3.1 (1.6,6.1)***	2.2 (0.9,5.3)
Primipara	53	24 (*45*)	29 (*55*)	1	1
Information on benefits of breastfeeding received during pregnancy					
Yes	177	119 (*67*)	58 (*33*)	2.9 (1.1,8.1)*	2.5 (0.7,9.2)
No	17	7 (*41*)	10 (*59*)	1	1
Most family and/or friends breastfeed					
Yes	72	55 (*76*)	17 (*24*)	2.3 (1.2,4.5)**	1.4 (0.6,3.3)
No	122	71 (*58*)	51 (*42*)	1	1
Half of the family and/or friends breastfed other half give their infants formula milk					
Yes	40	15 (*38*)	25 (*62*)	0.4 (0.2,0.7)**	0.3 (0.1,0.8)*
No	154	111 (*72*)	43 *(28*)	1	1
Awareness on how family and friends feed their young children					
Not aware	31	15 (*48*)	16 (*52*)	0.4 (0.2,0.96)*	0.8 (0.3,2.3)
Aware	163	111 (*68*)	52 (*32*)	1	1
Aware of having been breastfed as an infant herself					
Mother have been breastfed as infant	67	52 (*78*)	15 (*22*)	2.5 (1.3,4.9)***	1.9 (0.8,4.5)
Mother has not been breastfed as infant or don’t know	127	74 (*58*)	53 (*42*)	1	1
Intention while pregnant to breastfeed					
Yes	128	99 (*77*)	29 (*23*)	5.1 (2.7,9.9)***	4.0 (1.9,8.4)***
No	66	27 (*41*)	39 (*59*)	1	1
Employment					
Self-employed	109	79 (*72*)	30 (*28*)	2.1 (1.2,3.9)**	2.1 (1.0,4.7)
Not self-employed	85	47 (*55*)	38 (*45*)	1	1
Nipple pain					
Yes	18	6 (*33*)	12 (*66*)	0.2 (0.1,0.7)*	0.3 (0.1,0.9)*
No	176	120 (*68*)	56 (*32*)	1	1

Primarily breastfeeding: Children under 6 months of age who are fed with breast milk only and children older than 6 months of age that were exclusively breastfed up to six months, based on recall from birth

***P-value < 0.001, **0.001 ≤ *P* < 0.01, *0.01 ≤ *P* < 0.05 *COR* Crude Odds Ratio, *AOR* Adjusted Odds Ratio

In addition, we found a significant association between the parity of the mother and primarily breastfeeding. Seventy-two percent (102/141) of the (grand) multiparas practiced primarily breastfeeding, while only 45% (24/53) of the primiparas did so (COR 3.1; 95% CI 1.6, 6.1, *P* < 0.001, Table 3).

**Fig. 1:**
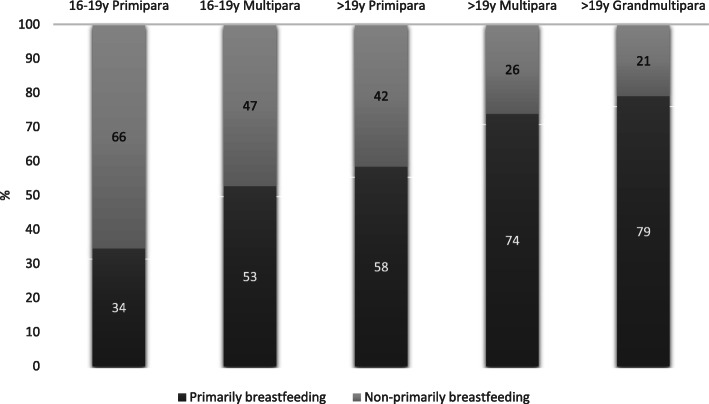
Distribution of primarily breastfeeding practice proportions by maternal age and parity

Of all the primipara mothers, 55% (29/53) were teenagers. Although fewer teenage primiparas practiced exclusive breastfeeding (10/29; 34%), the primarily breastfeeding rate of primipara mothers older than 19 was also lower (14/24; 58%) than that of (grand) multiparas, as shown in Fig. 1.

During the FGDs some of the women talked about their experiences as multigravidas:

*“I suffer the act of giving hot water to my children, but when I began to do exclusive breastfeeding for six months, I saw the difference: my child is very strong.”* (Community member, Bandajuma).

#### Assistance by HCW on early initiation of breastfeeding

In total 147 mothers (76%) had received assistance for the positioning of the baby to the breast by an HCW during the first breastfeeding, although not all the 147 gave breastfeeding within the first hour. Of those mothers who gave breastfeeding within 1 h, 80% (118) were assisted by a Health Care Worker. While women who did not breastfeed within 1 h, 47% (26) were not assisted by 81 % (112) of the 138 mothers who delivered in a health center were assisted by an HCW, while only 65% (35) of the 54 mothers who delivered in the hospital were assisted by an Health Care Worker. During the interviews and FGDs mothers also mentioned that they received assistance from the HCW with the early initiation of breastfeeding:

*“When I gave birth, after the cord was cut, the nurse, who was my aunt, took my baby and placed him on my chest to breastfeed him. She assisted me.”* (Mother, Sahn Malen, 27 years).

HCWs saw it as part of their role to assist women with their first breastfeeding.

*“To put the baby to the breast. That is my role.”* (Nurse)*.*

Hospital midwives also saw promoting breastfeeding as part of their job; however, they faced challenges in doing so due to time constraints.

*“Yes, we try, when they are in the ward, when we have time*. *.. They spend less time with us and most of the time we tend to forget to talk to them about breastfeeding. They deliver, and they are fine.”* (Hospital HCW).

In addition, 86% (150/175) of women who had vaginal deliveries breastfed their infants within the first hour, while only 53% of women who delivered by Caesarean section did so.

#### Provision of information

Table 3 shows the odds ratio of factors significantly associated with primarily breastfeeding, including factors related to the provision of information to women. Antenatal care visits provide the pregnant women with an opportunity to receive information on child feeding. As mentioned before, 182 women (94%) reported having received information during their pregnancies about the feeding of infants of which most of the women (178, 92%) received this information at the health center. A slightly lower number (177, 91%) received specific information on the health benefits of breastfeeding. Providing information alone is not a factor significantly associated with primarily breastfeeding, while receiving *specific* information on the benefits of breastfeeding is significantly associated with primarily breastfeeding practice (COR 2.9; 95% CI 1.1, 8.1, Table 3). Examples of the information provided are shown in Table 4, as described by mothers during the interviews and FGDs. Qualitative data show that HCWs do not refer to the counselling cards available at all health facilities as a tool to provide information. Almost all of the women (181/194; 93%) reported having heard about breastfeeding on the radio:

*“They advise us [on the radio] about exclusive breastfeeding for six months and taking good care of our babies.”* (Mother, Bandajuma, FGD).

**Table 4:** Information provided at the health center on exclusive breastfeeding (EBF)

• Breastfeeding is good.	
• Practice EBF for 6 months.	
• Your baby will become healthy, strong, plump and intelligent.	
• Your baby will not become malnourished.	
• Your baby will not get sick.	
• Do not nourish your baby with anything other than breast milk.	
• Do not give your baby hot water, native herbs or other foods.	

During the FGDs, fathers, family and other community members also mentioned having received information on breastfeeding via the radio*:*

*“Yes, we get a lot of learning from the radio and hospital that breastmilk is good for babies.”* (Father, Bandajuma).

#### Influencers on decision-making

This study reveals that HCWs greatly influence the decision-making process of mothers around child feeding. Fifty-six percent (108/194) of the mothers in our study indicated that an HCW helped them with breastfeeding. The qualitative data showed that a large majority of the mothers were influenced by an HCW on breastfeeding practice (Table 5). Not only HCWs, but also community health workers (CHWs), provide information on breastfeeding in the villages. While many mothers stated they had received information from HCWs at the health center, some fathers and community members did so from CHWs:

*“We have CHWs who are also helping to inform us.”* (Father Bandajuma).

**Table 5:** Themes and illustrative quotations related to breastfeeding determinants

Theme	Quotes
**HCWs’ influence mothers’ feeding decisions**	“*It was the nurse* [who influenced me regarding the way I fed my baby].” (Mother, Pujehun, 23 years) *“After two weeks she was just crying, I wanted to give her* [powder] *milk but the nurse advised me* [not to].” (Mother, Sahn Malen, 26 years). *“The nurses* [had me feed my baby this way.]*”* (Mother, Pujehun, 33 years)
**Husbands’ involvement with child feeding practices**	*“The father of the baby* is supporting me *because he gives me enough food. He encourages me*.” (Mother, Sahn Malen,22 years) “*My husband supports me, for example by providing food for me.”* (Mother, FGD, Bandajuma) “*We fathers should support lactating mothers by giving them enough food so that they can feed our babies well. Encouragement is also important, because without encouragement the woman will find it difficult to breastfeed her baby.”* (Father, Pujehun) *“Yes, if you are a farmer, businessman or carpenter, just work hard to provide food for your wife. If not, she will not breastfeed your baby exclusively and she will go out for food. When your wife is without food, she will disobey you.”* (Father, Bandajuma) *“Most people used to say that a newborn given exclusive breastfeeding will feel hungry, and sometimes they feed babies hot water, but now I have realized that is not true, that the best food* *for a baby is breastmilk. In fact, giving hot water to baby will result to cough.”* (Father, Pujehun) *“It was his father who decided to give the baby hot water, because her stomach kept drying, so he told me to give her hot water one morning and after that the child has passed stool.” (*Mother, Zimmi, 26 years) *“Most women also refused to breastfeed their babies because of lack of encouragement from their husbands. Today most young husbands do not take care of their wife, they will neglect them for another woman because they are lactating mothers”* (Mother FGD, Bandajuma)
**Issues of sex and breastfeeding**	*“[No sex] For the simple reason that it will affect the breastmilk, and if you feed your baby with such breastmilk the baby will end up malnourished. Also, just having sex could lead to another pregnancy, which would mean there is not enough time to care for the baby properly. For me, whenever my wife is breastfeeding, I go out.”* (Father Pujehun) *“I heard a mother went to have sex with someone else to get food and then her child became ill.”* (Father Bandajuma)
**Mothers’ reasons for feeding items alongside breastmilk before the child reaches 6 months of age**	*“When he was 3 weeks old [I started giving him other food]. That is because my breast was not having enough breast milk.”* (Mother Pujehun, 20 years) *“Because there are times that the breast milk was not enough for him.”* (Mother, Bandajuma, 23 years) *“Her stomach was dry; it kept drying. She could not pass stools.”* (Mother, Zimmi,26 years) *“Because the baby is always crying, I give it hot water.”* (Mother, Bandajuma, 20 years) *“Lack of enough food for the mother; if the mother is not satisfied, she can hardly feed her baby.”* (Mother, Pujehun FGD) *“Except if I am hungry. If I am hungry, I will not be able to breastfeed my child because my head will start to spin.’* (Mother, Gbondapi, 40 years).

Although the qualitative data revealed that mothers and community members received information on EBF from traditional birth attendants (TBAs), the qualitative results revealed no major influence of TBAs over women’s decision-making with respect to primarily breastfeeding. Health Care Workers mentioned that TBAs could be helpful in disseminating information; however, they also stated that some TBAs do not encourage women to go to health facilities.

*“They are very important, TBAs and CHWs. Because they live with them [breastfeeding mothers]. They are on the front line. They know everything about them. They listen to them. Some things that they [breastfeeding mothers] will not share with us, they will tell them “. (*HCW, Pujehun).

In addition to HCWs, husbands also greatly influence women’s decision-making on breastfeeding practices. Nineteen percent of the women (37/194) indicated that their husbands had supported them to continue breastfeeding. Of these women who are supported by their husband, 78% (24/37) of mothers practiced primarily breastfeeding. Furthermore, 9% (6/68) of the mothers who did not practice primarily breastfeeding for the first 6 months of their infants’ lives indicated that their husbands had influenced them in deciding to stop breastfeeding. During the SSIs, a quarter of the mothers stated that they were supported by their husbands, mentioning examples such as provision of food and encouragement. The fathers themselves saw it as a husband’s role to provide food and encourage one’s wife (Table 5). Qualitative data revealed that many respondents believed that having sex during breastfeeding would contaminate a woman’s breast milk and lead to malnutrition in her infant (Table 5). None of the single mothers (*n* = 11) practiced primarily breastfeeding, with a correlation coefficient of − 0.33 (not statistically significant).

The mothers mentioned the influence of other family members and friends to a lesser extent. Four percent (8/194) of all mothers stated that they were supported by their families, and 10% (7/68) of the mothers who were not practicing primarily breastfeeding indicated that family members had influenced them to stop exclusive breastfeeding. In line with these results, qualitative data also show that family influence can be both a contributing factor and a barrier in terms of exclusive breastfeeding*.*

*“Family, my mother. She used to tell me that people were saying that breast milk is very good for the baby and it is good that every mother should breastfeed her baby from birth to six months.”* (Mother Gbondapi, 29 years).

*“When my baby was four months old my aunty told me to try some other food. Corn milk.”* (Mother Pujehun, 26 years).

*“At first somebody in the community will come and just say there is bad water in the baby’s stomach, and the only thing that will remove the bad water is hot water.” (*Community member, Bandajuma).

The knowledge and the ways in which a mother’s family and friends fed their babies was significantly associated with her own breastfeeding practice. There is an independent negative association in terms of the woman’s own primarily breastfeeding practice if some woman’s family members and friends breastfed and some and gave their babies infant formula (AOR 0.3; 95% CI 0.1, 0.8; *P* < 0.1, Table 3). If a mother was unaware of what her family members and friends were feeding their children, there was the same negative association (COR 0.4; 95% CI 0.2, 0.96; *P* < 0.05, Table 3). Mothers’ awareness of having been breastfed as babies themselves was associated with primarily breastfeeding practice (COR 2.5; 95% CI 1.3, 4.9; *P* < 0.001, Table 3). Fifty-one percent of all the mothers surveyed did not know how they had been fed as babies.

#### Attributes of the mothers

We found a significant independent association between a mother’s intention to breastfeed and her primarily breastfeeding practice (AOR 4.0; 95% CI 1.9, 8.4; *P* < 0.001), Table 3). Of the mothers surveyed, 66% (128/194) stated that they intended to breastfeed their children, when they were pregnant. Of the women who intended to breastfeed, 77% (99/128) ended up practicing primarily breastfeeding. This is comparable with the qualitative data, where slightly more than half of the mothers interviewed indicated that they intended to breastfeed.

Despite the influence of other people on maternal decision-making, a majority of the mothers who stopped EBF prior to 6 months indicated that the decision was their own (41/68, 60%).

“*The baby cried a lot and I tried to breastfeed her, but she did not accept the breast milk, so I boiled a little bit of hot water and gave it to her. After that she fell asleep. It is my own experience that influenced me.”* (Mother, Gbondapi, age above 19 years).

*“I decided myself. .. They were all advising me to breastfeed my baby and no one was supporting me about giving corn milk or rice pap.”* (Mother, Sahn Malen, 19 years).

#### Workplace and employment

Although the majority of the surveyed women indicated that it was possible to breastfeed their babies during work, FGD participants suggested that women experience difficulties practicing EBF while farming.

*“They don’t have time to sit and breastfeed their babies. Sometimes when farming, the baby will be in the hut while they are busy working on the farm. So, most of the time they give enough strong food (*such as corn milk or a porridge made of rice, beans, fish, and sesame seeds or groundnuts, homemade or pre-packed) *so that they will have time to do their farm work.”* (Mother, Bandajuma FGD).

*“Also, it is difficult for those who work on farms to go to the hut repeatedly, so they give hot water to their babies so they will sleep, and the mother will have time to work on the farm.”* (Mother, FGD, Bandajuma).

*“Some women start weaning at two months and start giving pap. If you give pap, the child will sleep more so you have more time to work.”* (HCW, Pujehun FGD).

A mothers being self-employed was positively associated with primarily breastfeeding (COR 2.1; 95% CI 1.2, 3.9, *P* 0.01). Although being a student was not statistically significantly associated with breastfeeding practice, Focus Group Discussion participants suggested that mothers who attended school found it difficult to breastfeed.

*“Some lactating mothers attend school, so most of the time they leave their babies at home with their parents, and if they are crying the only thing, they can do is give them milk or hot water.”* (Mother, Bandajuma FGD).

### Reasons given for feeding babies something other than breastmilk before they turn six months old

Our quantitative data showed that one of mothers’ major reasons for giving their babies something other than breastmilk was their perception that the babies felt hungry (31%) or were unwell (31%).

The qualitative data revealed that a major barrier to mothers’ continuing to practice EBF was their perception that they were producing insufficient breast milk and that their baby’s stomach was “dry” (meaning that its stools were infrequent). Other frequent reasons given by mothers were that their baby was crying and/or that they themselves were hungry because of a lack of food (Table 5). A factor mentioned by HCWs, though not confirmed by the mothers, was that some women do not practice EBF due to a belief that babies who are breastfed alone get worms. In addition, difficulties with breastfeeding were mentioned as a barrier to practicing exclusive breastfeeding. This was confirmed by quantitative data: 9 % (18/194) of the women reported having had problems with nipple pain, and two-third of these mothers did not practice primarily breastfeeding all the way through their baby’s first 6 months. Experience of nipple pain was independent negatively associated with primarily breastfeeding (AOR 0.3; 95% CI 0.8, 0.9, *P* < 0.05, Table 3).

## Discussion

Under-five morbidity and mortality in Sierra Leone is still alarmingly high. Practicing EFB and appropriate complementary feeding contribute to prevent child mortality and positively affects children health, nutrition and developmental outcome [1, 18]. Improving the nutritional status of children is a priority in the strategic plan to decrease the mortality rate in Sierra Leone [18], and understanding breastfeeding practice determinants is essential in order to devise appropriate strategies to promote EBF practice and improve child health.

The present study revealed an increased EBF rate in Pujehun District, compared to national surveys data of 2013 and 2019 [8, 9]. However, this study also shows a sharp decrease of EBF for children in age group 4–5 months. These results underscore the need for continues interventions in order to improve EBF practices to improve child health. This study suggest that interventions might be need starting from the antenatal care throughout the first 6 months of life of the newborn and at healthcare level as well as community level.

In this study, almost 30% of the mothers were teenage mothers and 27% were primipara. Our study revealed that teenagers and primipara were at-risk groups for non-EBF practice. These findings were similar to findings in other developing countries [19], and underscore the need to target teenagers and primigravida in tailor-made breastfeeding promotion activities. Further investigation will be necessary to establish whether teenagers experience different barriers from non-teenagers, for example school related barriers, something that this study suggests. However, breastfeeding promotion activities should not exclude multigravida, given the fact that not all multigravida practice exclusive breastfeeding.

This study highlighted that the HCWs present in health centers are an important source of information for mothers on child feeding: providing women with information on the health benefits of breastfeeding during pregnancy and the intention of women during pregnancy to breastfeed are both positively associated with exclusive breastfeeding. The women in our study indicated that nurses heavily influenced their breastfeeding practices, confirming, as shown in previous studies [15], that they play an important role in women’s decision making regarding pregnancy, delivery and the first few months of the child life.

As this study demonstrates, 71.6% of the mothers said that they started breastfeeding within 1 hour after their child’s birth, showing an increase from the 2013 figure of 57.8% and similar to the data from 2017 (75%) [8, 11]. Interestingly, of the women who breastfed within 1 hour after their children’s births, 80% received assistance from an HCW, while of the women who did *not* breastfeed within that first hour, 47% did not receive assistance from an HCW. This study suggests that there are still opportunities to improve mothers’ initiative to breastfeed their infants within 1 hour of their births and EBF practice till 6 months by improving the care which HCW are providing during pregnancy, post-delivery care and the first 6 months of life of the newborn.

Not only nurses have a role to play in promoting breastfeeding practice, so do CHWs, by providing information to fathers and other village stakeholders. As this study shows, and in line with a previous study in Sierra Leone [15], also fathers/husbands are major influencers regarding women’s child-feeding practices, in fact the majority of the women in our study who felt supported by their husbands practiced exclusive breastfeeding. However, some husbands influence women to discontinue exclusive breastfeeding. Expanding the work of CHWs in promoting best breastfeeding practice among husbands might be a good strategy for better equipping the latter in terms of their involvement with their wives’ child-feeding decisions. As well as promoting talking on breastfeeding in women’s groups, as mother’s knowledge that her family members and friends breastfed their children had a positive influence on her own breastfeeding practice, as well as the awareness of having been breastfed as a baby herself. These findings also has been found in other developed and countries [18, 19] and suggest that talking to the entire community about the benefits of breastfeeding might have a positive influence on women’s practice of exclusive breastfeeding.

Although nurses, husbands, and other family members or friends are breastfeeding influencers, the majority of the mothers who participated in this study stated that they made their own decision to stop breastfeeding. In our study, nipple pain, described as an individual determinant, was negatively associated with primarily breastfeeding, as confirmed by literature [19, 20]. Since breast complications could be prevented by teaching mothers the correct position and attachment methods, or can be reduced by expressing breastmilk or treated with antibiotics in case of severe infection of the breast [21, 22], it may be possible to overcome this barrier by e.g. early recognizing and treatment of the problem by the HCW and/or providing adequate information on prevention and treatment of breast complications toward the mothers. Another factor related to breastfeeding practice is occupation. Although the questionnaire indicated the women are more likely to practice primarily breastfeeding when self-employed compare to other occupations, participant of the FGD revealed that working on the farming might be a barrier for EBF practice. Qualitative data did not specify between different kind of self-employment.

Interesting, women gave several reasons for having started complementary feeding before 6 months. One major reason was their perception that their babies were hungry, and/or that they were not producing sufficient quantities of breastmilk, or that their baby was crying. A systematic review of factors influencing EBF in developing countries has shown that these barriers are found in several other developing countries [19]. It has yet to be determined whether the perceptions of mothers about not having sufficient breastmilk is related to their actual breast milk production, but it could be that there are other factors related to this perception, and breastfeeding counselling might help alter it. Counselling on correct breastfeeding techniques and on taking enough time to breastfeed infants might contribute, for example, to improved breast milk production [22]. Another reason provided by mothers for giving their babies something in addition to breast milk (mainly boiled water and herbs) was their perception that their babies were having “bad” stools. Participants of the interviews and FGDs referred to black meconium and infant constipation. There is no evidence that mothers’ perception of their babies’ constipation was related to actual medical issues; it could instead have been due to their inadequate knowledge of infant stool patterns. The stools of infants can be irregular and have a variable appearance [23]. Further investigation is needed to establish whether the infants believed by their mothers to have stool difficulties were actually suffering from constipation or whether that was merely the mothers’ perception. It is essential to provide information to mothers and community members on meconium and “normal stools” in order to try to prevent babies from being fed water and herbs early in life.

The study was subject to limitations. One major limitation of the design was that it was a facility-based survey, therefore the study was representative for the population visiting the health center only, as was described in the objective of the study, and could be associated with a higher health promotion exposure and higher EBF rate among this population compared to the general population. Despite this the high institutional delivery (90.2%), antenatal visits (98.8%) and immunization rates (96.5% for Pentavalent 3 and 96.5% for OPV3) in Pujehun District [11], could indicate that the population visiting the facility is representative for the general population. Another limitation of the study was that it was carried out in a single district of Sierra Leone, and might not be able to be extrapolated to the entire country. Although the EBF rate was calculated on 24-h recall, the statistically significant factors related to primarily breastfeeding and determinants revealed in the qualitative research were based on recall from birth, which may have introduced a recall bias.

## Conclusions

In conclusion, although the exclusive breastfeeding rate in Pujehun District is not yet optimal, it has grown over the years. However, the sharp decrease in the EBF rate by age group shows that more effort is needed to promote exclusive breastfeeding practice for all newborns under the age of 6 months.

This study revealed several barriers and enabling factors which can be taken into consideration to design new strategies to improve child health. Breastfeeding promotion campaigns focusing on specific messages adapted to the local context and beliefs can be a solution to avoid premature and unjustified interruption of breastfeeding practice. Furthermore, receiving information during pregnancy on the benefits of EBF, awareness on how family and friends feed their babies, support from husbands and counselling by the nurses are factors associated with breastfeeding practice. It therefore seems relevant to include health workers and family members in promotion programs to have a greater adherence of mothers to breastfeeding.

## Data Availability

The datasets used and analyzed during this study are available from the corresponding author on reasonable request.

## Abbreviations

ANC: Antenatal Care
CHW: Community Health Worker
CI: Confidence Interval
EBF: Exclusive Breast Feeding
FGD: Focus Group Discussion
HCW: Health Care Worker
IYCF: Infant and Young Child Feeding
OPV: Oral Polio Vaccination
PNC: Postnatal Care
SD: Standard Deviation
SLDHS: Sierra Leone Demographic Health Survey
SSI: Semi-structured Interview
TBA: Traditional Birth Attendant
WHO: World Health Organization
